# The Effect of Anticoagulants on Early Implant Failure: A Retrospective Cohort Study

**DOI:** 10.3390/jfb14040186

**Published:** 2023-03-27

**Authors:** Liat Chaushu, Noa Perez, Daniele Botticelli, Samuel Porfirio Xavier, Roni Kolerman, Daya Masri

**Affiliations:** 1 Department of Periodontology and Implant Dentistry, The Maurice and Gabriela Goldschleger School of Dentistry, Tel Aviv University, Tel Aviv 6934206, Israel; noap180195@gmail.com (N.P.); kolerman@netvision.net.il (R.K.); 2 ARDEC Academy, Viale Giovanni Pascoli 67, 47923 Rimini, Italy; daniele.botticelli@gmail.com; 3 Department of Oral and Maxillofacial Surgery and Periodontology, Faculty of Dentistry of Ribeirão Preto, University of São Paulo, Av. do Café-Subsetor Oeste-11 (N-11), Ribeirão Preto 14040-904, Brazil; spx@forp.usp.br; 4 Department of Oral and Maxillofacial Surgery, Rabin Medical Center, Beilinson Campus, Petah Tiqwa 49414, Israel; dr.dayamasri@gmail.com

**Keywords:** anticoagulants, dental implant, early implant failure

## Abstract

Background: Anticoagulants (AC) are among the most often prescribed drugs in the world. Data regarding ACs’ effect on the osseointegration of dental implants is lacking. Purpose: The aim of the present retrospective cohort study was to evaluate the effect of anticoagulants (AC) on early implant failure (EIF). The null hypothesis was that the use of AC increases the incidence of EIF. Materials and Methods: The research included 687 patients who underwent 2971 dental implant placements in the department of oral and maxillofacial surgery in Rabin medical center, Beilinson hospital, by specialists in oral and maxillofacial surgery. The study group comprised 173 (25.2%) patients and 708 (23.8%) implants using AC. The rest of the cohort served as a control. A structured form was used to collect data at patient and implant level. EIF was defined as implant failure within a period of up to 12 months from loading. EIF was the primary outcome parameter. A logistic regression model was used to predict EIF. Results: Implants placed in individuals ≥ 80 (odds ratio (OR) = 0.34, *p* = 0.05), and ASA 2/3 vs. ASA 1 individuals (OR = 0.30, *p* = 0.02/OR = 0.33, *p* = 0.03, respectively) had decreased odds of EIF, and implants in those using anticoagulants (OR = 2.64, *p* = 0.01) had increased odds of EIF. At the patient level, the odds of EIF in ASA 3 (OR = 0.53, *p* = 0.02) and IHD (OR = 0.40, *p* = 0.02) individuals decreased. In AF/VF (OR = 2.95, *p* = 0.01) individuals, EIF odds increased. Conclusions: Within the limitations of the present study, the use of AC is significantly associated with an increased likelihood of EIF: the OR was 2.64. Future research is required to validate and examine the prospective impact of AC on the osseointegration phenomena.

## 1. Introduction

Implant dentistry is a safe and current therapeutic option for patients who are totally or partially edentulous. Research into adverse events endangering osseointegration has appeared more and more in the existing literature [1]. Dental implants have a 10-year survival rate of 90–95 percent. Typically, failure occurs because of implant loosening, fracture, or infection. Adverse events may include pain, and on rare occasions, neuropathy [2].

The time of implant failure occurrence is used to categorize failures [2]. Early implant failure (EIF) occurs <1 year from abutment connection. Following occlusal loading, late implant (>1 year loading) failures occur. They are thought to develop as a result of a breakdown of established osseointegration [2].

Reports on the impact of systemic disorders on implant therapy may be found in the dental literature. Still, many oral and systemic diseases have unclear effects on osseointegration [3].

One of the most prevalent complications associated with endosseous implants is hemorrhage [4]. Anticoagulants (AC) are among the most often prescribed drugs in the world. They are generally used for patients who are at high risk of systemic, pulmonary, or cerebral embolism [3,4]. Patients with bleeding disorders may be more prone to complications. However, there is no convincing evidence that bleeding problems may hamper implant dentistry [3,4,5]. Whenever modifications of AC medications are discussed for surgery, the potential risks of postoperative bleeding on one hand vs. the potential for embolic events must be evaluated. In oral surgery, the risk of thromboembolic complications typically outweighs the risk of significant bleeding complications [3,4,5].

Extensive research has been conducted on the clinical effects of VKAs on human bone metabolism. There is evidence that VKAs affect bone metabolism. Long-term usage of oral AC has been related with an increased risk of osteoporosis. As a VKA, warfarin has a detrimental effect on bone health. Vitamin K is essential for osteocalcin’s posttranslational glutamination, the primary non-collagenous bone matrix protein. Dietary limitations typically undertaken by patients receiving VKAs are one additional potential indirect mechanism of bone deterioration [6,7].

There has only been a limited amount of clinical research on the effects of DOACs on bone since they are still relatively new to clinical practice. The possible effect that DOACs may have on bone metabolism have been poorly investigated so far [8,9]. In 2012, Gigi et al. studied the effects of DOACs on bone biology utilizing a human female osteoblastic cell culture model in vitro. Rivaroxaban dose-dependently reduced up to 60% of the cell’s DNA production. Creatine-kinase-specific activity was likewise dose-dependently decreased to a comparable degree, whereas alkaline-phosphatase-specific activity was suppressed by up to 30%. Osteoblastic mineralization was unaffected, showing that rivaroxaban suppresses the initial stage of bone formation but has no influence on later stages (mineralization of bone), resulting in a transitory suppression of bone development [10]. According to several studies, rivaroxaban therapy may have an adverse effect on bone by reducing osteoblastic function. The osteocalcin bone marker, the transcription factor Runx2, and the osteogenic factor bone morphogenetic protein (BMP)-2 all showed decreased mRNA expression in conjunction with this reduction [11]. According to a study conducted in a population of Hong Kong patients with non-valvular AF, Dabigatran was related to a significantly lower risk of osteoporotic fracture than Warfarin [12]. DOACs are associated with a lower influence on bone metabolism and potentially a decreased risk of fractures compared to VKAs, according to the existing data [13,14].

The aim of the present retrospective cohort study was to evaluate the effect of anticoagulants (AC) on EIF. The null hypothesis was that use of AC increases the incidence of EIF.

## 2. Method and Materials

The research included 687 patients who underwent 2971 dental implant placements in the department of oral and maxillofacial surgery in Rabin medical center, Beilinson hospital, by specialists in oral and maxillofacial surgery. Early implant failure (EIF) was defined as the removal of the implant from the oral cavity within a period of up to 12 months from the date of its loading in the oral cavity.

Inclusion criteria:

- Patients who underwent dental implant placement in the department of oral and maxillofacial surgery in Beilinson hospital.
- Follow-up, ≥12 months from the date of implant loading.
- Each patient had a CT scan before the transplant.
- Follow-up included X-rays (panoramic), and clinical examination.

Exclusion criteria:

- Follow-up of at least <1 year from loading.
- Severe systemic diseases that include immunosuppression.
- Patients who underwent head and neck radiation ≥ 5000 rad.

Variables collected at implant level included:

- Age Group—≤65 or 66–79.9 or ≥80.
- Physical status—ASA 1/ASA 2/ASA 3 [15].
- Smoking status—No/Yes.
- Use of AC.
- Systemic diseases—HTN; DM; CVA; hyperlipidemia.
- Implant brand—Ditron Dental/MIS/Zimmer Dental.
- Bone augmentation—Pristine/Augmented.
- Implant location—maxilla/mandible; anterior/premolar/molar.
- Implant dimensions—length, diameter.
- EIF—primary outcome parameter.

Variables collected at patient level included:

- Gender—male/female.
- Age Group—≤65 or 66–79.9 or ≥80.
- Physical status—ASA 1/ASA 2/ASA 3.
- Smoking status—No/Yes.
- Use of AC.
- Systemic diseases—IHD; AF/VF.
- Bone augmentation—Pristine/Augmented.
- Total implant number placed per individual.
- EIF—primary outcome parameter.

## 3. Statistical Analysis

The software SPSS version 25 was used for data analysis. Tests conducted were as follows: Chi-square ($\chi^{2}$)—univariate correlations; Mann-Whitney—independent samples. A *p*-value < 0.05 was considered statistically significant.

## 4. Results

### 4.1. Demographic Data

The cohort (n = 687) comprised 61.7% males and 38.3% females. Ages (years) were: 58.2% ≤65 years, 32.5% 66–79.9 years, and the remaining 9.3%, ≥80 years or older. Physical status was: 32.5%—ASA 1; 35.4%—ASA 2, and the remaining 32.2%—ASA 3. A quarter (173/25.2%) used anticoagulants. Systemic diseases included: 12.2%—ischemic heart disease (IHD); and 8.9%—atrial/ventricular fibrillation (AF/VF). Smokers made up 5.4%. Implant brands were: 12.5%—Ditron Dental; 27.4%—MIS; and 60.1%—Zimmer Dental. More than half (55.7%) of the individuals required bone augmentation for implant placement. The EIF cohort at patient level was 15.1%.

All data were likewise analyzed at implant level (n = 2971). Ages were 57.7% ≤65 years, 34% 66–79.9, and the remining 8.3% ≥80 or older. Physical status was 27.3%—ASA 1, 39.6%—ASA 2, and 33%—ASA 3. A quarter (708) of the implants (23.8%) were inserted in individuals using anticoagulants. Implants inserted in individuals with systemic diseases included: hypertension (HTN)—29%; hyperlipidemia—23.6%; DM—14.9%; and cerebrovascular accident (CVA)—4.6%. Implants inserted in smokers made up 6.6%. Implant brands were: 13.6%—Ditron, 26.3%—Mis, and 60.1%—Zimmer. Most of the implants (61.2%) required augmentation. The implant location was: 15.3% anterior maxilla, 18.5% premolar maxilla, 15% posterior maxilla, 13.7% anterior mandible, 17% premolar mandible, and 20.5% were for posterior mandible. Lastly, EIF was recorded for 3.8% of the implants. For a complete description, see Table 1 for implant level descriptive statistics and Table 2 for patient level descriptive statistics.

### 4.2. Variables

Table 3 and Table 4 describe data distribution.

## 5. Results

At implant level (Table 5), significant characteristics of not using AC included age groups ($\chi^{2}\left(2\right)=71.07,p<0.001$). Patients ≤ 65 or less were more likely to not use AC (62% vs. 44.2%). However, those between the ages of 66 and 67.9 and those over the age of 80 were more likely to use AC (43.8% and 12%, respectively, vs. 30.9% and 7.2%, respectively).

Smokers were more likely to use AC (8.6% vs. 5.9%) ($\chi^{2}\left(1\right)=6.38,p<0.001$). Hypertensive individuals (50.7% vs. 22.2%) ($\chi^{2}\left(1\right)=213.17,p<0.001$), those with DM (24.9% vs. 11.8%) ($\chi^{2}\left(1\right)=71.88,p<0.001$), those with CVA (9.3% vs. 3.2%) ($\chi^{2}\left(1\right)=45.91,p<0.001$), and those with hyperlipidemia (40% vs. 18.4%) ($\chi^{2}\left(1\right)=138.99,p<0.001$) were more likely to use AC.

A significant difference was found for the ASA group ($\chi^{2}\left(2\right)=648.47,p<0.001$). Individuals with ASA 1 with implants were more likely not to use AC (34% vs. 5.9%). Similarly, ASA 2 individuals with implants were more likely not to use AC (45.1% vs. 22.3%). However, those with ASA 3 who had implants were more likely to use AC (71.8% vs. 20.9%). Those with implants placed in the anterior maxilla were more likely not to use AC (16.3% vs. 12.1%) ($\chi^{2}\left(1\right)=7.19,p=0.007$).

Those with implants in the anterior mandible were more likely to use AC (18.9% vs. 12%) ($\chi^{2}\left(1\right)=22.13,p<0.001$). Those with implants in the posterior mandible were more likely not to use AC (21.4% vs. 17.1%) ($\chi^{2}\left(1\right)=6.29,p=0.01$). The overall implant length was increased for those using AC (11.51±1.50 vs. 11.34±1.63) (*p* = 0.05). EIF was more likely to occur in those using AC (5.5% vs. 3.3%) ($\chi^{2}\left(1\right)=7.04,p=0.008$).

At the patient level, a significant difference was found for gender ($\chi^{2}\left(1\right)=12.66,p<0.001$). More females did not use AC (65.5 vs. 50.3%), while males were more likely to use AC (49.7% vs. 34.5%). An additional significant difference was found for age groups ($\chi^{2}\left(2\right)=48.41,p<0.001$), those ≤65 were more likely to be without AC (65.6% vs. 36.4%), yet those between the ages of 66 and 79.9 and those ≥80 were more likely to use AC (46.2% and 17.3%, respectively) than not (27.8% and 6.6%, respectively). Additionally, differences were found for ASA ($\chi^{2}\left(2\right)=184.15,p<0.001$): ASA 1 and 2 individuals were more likely not to use AC (41.7% and 39.8%, respectively vs. 4.7% and 22.2%, respectively). However, ASA 3 individuals were more likely to use AC (73.1% vs. 18.5%). Those with IHD were more likely to use AC (31.2% vs. (5.8%) ($\chi^{2}\left(1\right)=77.47,p<0.001$). Those with AF/VF were more likely to use AC (21.4% vs. 4.7%) ($\chi^{2}\left(1\right)=44.71,p<0.001$). For full model tests, see Table 5 and Table 6.

## 6. Multivariate Analysis

A logistic regression model at the implant level showed that the independent variables significantly predict failure (*χ*^2^(15) = 33.07, *p* = 0.005), while it explains about 9.6% of total variance in failure. The model is well fitted to the data (*χ*^2^(8) = 2.78, *p* = 0.95), while it classifies about 96.3% of the total observations.

Implants placed in individuals ≥80 (OR = 0.34, *p* = 0.05), and ASA 2/3 vs. ASA 1 individuals (OR = 0.30, *p* = 0.02/OR = 0.33, *p* = 0.03, respectively) had decreased odds of EIF and implants in those using anticoagulants (OR = 2.64, *p* = 0.01) had increased odds of EIF. For complete regression coefficients, see Table 7.

A logistic regression model at the patient level showed that the independent variables significantly predict failure (*χ*^2^(9) = 27.12, *p* = 0.001), while they explain about 7% of total variance in failure. The model does not fit to the data well (*χ*^2^(6) = 8.31, *p* = 0.22), while it classifies about 85.0% of the total observations.

At patient level the odds of EIF in ASA 3 (OR = 0.53, *p* = 0.02) and IHD (OR = 0.40, *p* = 0.02) patients are decreased. In patients with AF/VF (OR = 2.95, *p* = 0.01), the EIF odds are increased. No statistically significant difference in EIF odds was found for those using AC (*p* = 0.87) while controlling for the other variables. For complete regression coefficients, see Table 8.

## 7. Discussion

The potential impact that AC may have on bone metabolism have been poorly investigated so far. The few existing research studies are inconsistent. The evidence from the literature on the effects of AC on bone health is heterogeneous, meaning that it is not consistent and may vary depending on the specific medication and the duration of treatment. Osseointegration is a complex process that is influenced by many factors, including the type of implant, the location of the implant, and the overall health of the individual. As such, it is important to carefully consider all of these factors when evaluating the potential effects of medications on osseointegration.

Various mechanisms have been postulated to explain how AC disrupt bone biology, and these mechanisms may be extrapolated to explain how these drugs may impede osseointegration. However, it is important to note that these results may not necessarily be applicable to humans and further research is needed to determine the effects of AC on osseointegration in people.

According to a series of in vitro investigations conducted by Gigi et al. [10]. Rivaroxaban induces a decrease in osteoblastic cell proliferation and energy consumption, as well as a minor suppression of the osteoblastic marker, alkaline phosphatase, but osteoblastic mineralization remains unchanged. This is a phenomenon that can be explained by the action of the medicine being temporary or by the cells adapting to the inhibitory effect and compensating via another method. Furthermore, they revealed that rivaroxaban inhibits the modulation of bone cells by several hormones, including estrogenic compounds, vitamin D compounds, and PTH. The mechanisms for the stimulatory actions of bone-modulating hormones remain unknown. To summarize, these results suggest that rivaroxaban may prevent the first step in bone formation without affecting subsequent steps (i.e., bone mineralization).

Treatment with rivaroxaban and enoxaparin [11] decreased alkaline phosphatase activity, and the expression of bone morphogenetic protein (BMP)-2, osteocalcin, and Runx2. The conclusion implied a negative outcome on osteoblast activity. Moreover, prolonged therapy with those drugs could jeopardize bone homeostasis [16,17]. Others [18] demonstrated an increased callus volume with lower density following fracture when those drugs were used. This phenomenon was associated with poor fracture healing [19]. A possible explanation is the creation of a bigger hematoma following fracture using anticoagulant therapy [18].

Research examining the potential impact of rivaroxaban on osseointegration demonstrate that rivaroxaban does not have a detrimental effect on osseointegration in this model [20].

In the present study, the effect of AC on EIF was investigated. Logistic regression was used to analyze potential predictors of EIF in a sample of dental implants. Two logistic regression models were conducted, at the implant and the patient level. The results suggest that certain independent variables are related to the likelihood of EIF. At the implant level, the use of AC was found to be associated with increased odds of EIF. However, at the patient level, no significant relationship was found between anticoagulant use and implant failure when controlling for other variables.

We also observed that patients with a higher American Society of Anesthesiologists (ASA) score (ASA 2 or 3) had lower odds of implant failure than those with an ASA 1 score. These data imply that, depending on the ASA classification, there might be protective factors or other mechanisms that influence the probability of EIF. The treatment and management of patients with a higher ASA score may have been more thorough, with a greater emphasis on avoiding risks and optimizing outcomes, which may explain this finding [21]. Further research is required to completely comprehend the mechanisms underlying this association and to establish the optimal strategy for decreasing EIF in patients with varying ASA scores.

It was found that EIF odds for patients with IHD were lower. Patients with AF/VF had increased EIF. Patients with serious medical conditions are often managed with medications and lifestyle changes to reduce the risk of complications. These interventions may also help to reduce the risk of EIF. It is probable that this is reflected in the data for patients with IHD. It is also worth noting that there may be other factors that contribute to the relationship between IHD and EIF, or between AF/VF EIF. To better understand these relationships and to identify the most effective strategies for reducing implant failure in patients with different medical conditions, it will be important to conduct additional research and to consider a wider range of factors that may be influencing the outcome.

One of this study’s limitations is not measuring bone densitometry due to its retrospective nature. Future studies should take this factor into account. There is also the possibility of bias: for patients who take oral anticoagulants, this is usually due to having a cardiac arrhythmia such as atrial fibrillation. This means older age, higher rates of hypertension, and possibly, smoking, factors that also affect bone remodeling.

Osseointegration was compared between 80 implants with hydrophilic SLActive and hydrophobic SLA surface in 20 anticoagulated patients. A “split-mouth” study design was used. After one year, 100% implant survival and success rates were observed [22].

A retrospective, study (297 implants, follow-up of up to 76 months) assessed anticoagulant drugs’ effect. A 4.4% lower survival rate was reported, and the odds ratio of failure was 28.2. It was concluded that anticoagulants increase the risk of implant failure [23].

A recent review revealed that non-steroidal anti-inflammatory drugs, glucocorticoids, proton pump inhibitors, selective serotonin reuptake inhibitors, anticoagulants, metformin, and chemotherapeutic agents may jeopardize osseointegration and lead to implant failure. However, validation is mandatory from human studies with high evidence [24].

The results of the present study suggest that the risk of EIF may be influenced by numerous factors. Additional research is required to better understand the mechanisms underlying these associations and to identify strategies for reducing the risk of EIF. This may entail replicating the study in a different sample, examining the role of other patient characteristics and treatment factors, and investigating the mechanisms underlying the associations between medical conditions and implant failure.

## 8. Conclusions

Within the limitations of the present study, the use of AC is significantly associated with an increased likelihood of EIF. The OR was 2.64. Future research is required to validate and examine the prospective impact of AC on the osseointegration phenomena.

## Figures and Tables

**Table 1:** Demographic and baseline clinical characteristics of the cohort at implant level (n = 2971).

	M	SD	N	%
**Demographic Characteristics**				
Age group (years)				
≤65			1715	57.7
2. 66–79.9			1009	34
3. ≥80			247	8.3
Physical status				
ASA 1			812	27.3
2. ASA 2			1178	39.7
3. ASA 3			981	33.0
Anticoagulants (AC)			708	23.8
Hypertension (HTN)			861	29
Hyperlipidemia			700	23.6
Diabetes mellitus (DM)			444	14.9
Cerebro vascular accident (CVA)			138	4.6
Smoking			195	6.6
**Clinical Characteristics**				
Implant brand				
Ditron			403	13.6
2. MIS			783	26.3
3. Zimmer			1785	60.1
Bone augmentation				
Pristine			1153	38.8
2. Augmented			1818	61.2
Implant location				
Anterior maxilla			456	15.3
2. Premolar maxilla			549	18.5
3. Posterior maxilla			446	15
4. Anterior mandible			406	13.7
5. Premolar mandible			506	17.0
6. Posterior mandible			608	20.5
Implant				
Length	11.38	1.60		
Width	3.85	0.42		
EIF			114	3.8

**Table 2:** Demographic and baseline clinical characteristics of the cohort at patient level (n = 687).

	M	SD	N	%
**Demographic Characteristics**				
Gender				
Male			424	61.7
2. Female			263	38.3
Age group (years)				
≤65			400	58.2
2. 66–79.9			223	32.5
3. ≥80			64	9.3
Physical status				
ASA 1			223	32.5
2. ASA 2			243	35.3
3. ASA 3			221	32.2
Anticoagulants			173	25.2
Ischemic heart disease (IHD)			84	12.2
Atrial/Ventricular fibrillation (AF/VF)			61	8.9
Smokers			37	5.4
**Clinical Characteristics**				
Implant brand				
Ditron			86	12.5
2. MIS			188	27.4
3. Zimmer			413	60.1
Bone augmentation			383	55.7
Total implants placed per individual	4.32	3.68		
EIF			104	15.1

**Table 3:** Study variables at implant level.

	Values	Normality Tests
Age Group	≤65 or 66–79.9 or ≥80	
Smoking	No/Yes	
HTN	No/Yes	
DM	No/Yes	
CVA	No/Yes	
Hyperlipidemia	No/Yes	
Implant type	Ditron/MIS/Zimmer	
Bone augmentation	Pristine/Augmented	
Physical status	ASA 1/ASA 2/ASA 3	
Anterior maxilla	No/Yes	
Premolar maxilla	No/Yes	
Posterior maxilla	No/Yes	
Anterior mandible	No/Yes	
Premolar mandible	No/Yes	
Posterior mandible	No/Yes	
Implant length		Non-normal; p<0.001
Implant width		Non-normal; p<0.001
Failure	No/Yes	
Anticoagulants	No/Yes	

**Table 4:** Study variables at patient level.

	Values	Normality Tests
Gender	Male/Female	
Age group	≤65 or 66–79.9 or ≥80	
Smoking	No/Yes	
Augmentation	No/Yes	
Physical status	ASA 1/ASA 2/ASA 3	
IHD	No/Yes	
AF/VF	No/Yes	
Total implants per individual		Non-normal; p<0.001
EIF	No/Yes	
Anticoagulants	No/Yes	

**Table 5:** Univariate tests at the implant level.

		No Anticoagulants		Anticoagulants			
Variable	Group	N (%)	M±SD	N (%)	M±SD	*p*-Value	
Age groups (years)	≤65	1402 (62)		313 (44.2)		<0.001	
	66–79.9	699 (30.9)		310 (43.8)			
	≥80	162 (7.2)		85 (12.0)			
Smoking	Yes	134 (5.9)		61 (8.6)		0.01	
HTN	Yes	502 (22.2)		359 (50.7)		<0.001	
DM	Yes	268 (11.8)		176 (24.9)		<0.001	
CVA	Yes	72 (3.2)		66 (9.3)		<0.001	
Hyperlipidemia	Yes	417 (18.4)		283 (40)		<0.001	
Bone augmentation	Pristine	875 (38.7)		278 (39.3)		0.78	
	Augmented	1388 (61.3)		430 (60.7)			
Physical status	ASA 1	770 (34)		42 (5.9)		<0.001	
	ASA 2	1020 (45.1)		158 (22.3)			
	ASA 3	473 (20.9)		508 (71.8)			
Anterior maxilla	Yes	369 (16.3)		86 (12.1)		0.007	
Premolar maxilla	Yes	427 (18.9)		121 (17.1)		0.29	
Posterior maxilla	Yes	334 (14.8)		111 (15.7)		0.55	
Anterior mandible	Yes	271 (12.0)		134 (18.9)		<0.001	
Premolar mandible	Yes	375 (16.6)		130 (18.4)		0.27	
Posterior mandible	Yes	485 (21.4)		121 (17.1)		0.01	
Implant length (mm)			11.34 ± 1.63		11.51 ± 1.50	0.05	
Implant diameter (mm)			3.85 ± 0.41		3.85 ± 0.44	0.40	
EIF	Yes	75 (3.3)		39 (5.5)		0.01	

**Table 6:** Univariate tests at patient level.

		No Anticoagulants		Anticoagulants		
Variable	Group	N (%)	M±SD	N (%)	M±SD	*p*-Value
Gender	Female	336 (65.5)		87 (50.3)		<0.001
	Male	177 (34.5)		86 (49.7)		
Age groups (years)	≤65	337 (65.6)		63 (36.4)		<0.001
	66–79.9	143 (27.8)		80 (46.2)		
	≥80	34 (6.6)		30 (17.3)		
Physical status	ASA 1	214 (41.7)		8 (4.7)		<0.001
	ASA 2	204 (39.8)		38 (22.2)		
	ASA 3	95 (18.5)		125 (73.1)		
IHD	Yes	30 (5.8)		54 (31.2)		<0.001
AF/VF	Yes	24 (4.7)		37 (21.4)		<0.001
Smoking	Yes	23 (4.5)		14 (8.1)		0.07
Bone augmentation	Yes	294 (57.2)		89 (51.4)		0.19
Total implants number per individual			4.34 ± 3.83		4.28 ± 3.19	0.30
EIF	Yes	72 (14)		32 (18.5)		0.15

**Table 7:** Binary logistic regression coefficients (at the implant level) to predict implant failure.

	OR	95% CI Lower	95% CI Upper	*p*-Value
Age group (years) (≥80)	0.34	0.12	0.99	0.05
Smokers	1.34	0.35	5.09	0.67
HTN	0.87	0.41	1.84	0.72
DM	1.02	0.44	2.40	0.96
CVA	0.84	0.19	3.81	0.82
Hyperlipidemia	1.00	0.47	2.12	0.99
ASA (ASA 2)	0.30	0.11	0.81	0.02
ASA (ASA 3)	0.33	0.12	0.88	0.03
Anterior maxilla	0.29	0.07	1.26	0.10
Anterior mandible	1.75	0.88	3.50	0.11
Posterior mandible	0.33	0.09	1.15	0.08
Implant length	1.07	0.86	1.33	0.55
Anticoagulants	2.64	1.31	5.32	0.01

Note: The reference group for the age group variable is “<80”. The reference group for ASA is “ASA 1”.

**Table 8:** Binary logistic regression coefficients (patient level) to predict EIF.

	OR	95% CI Lower	95% CI Upper	*p*-Value
Age group (years) (≥80)	0.65	0.41	1.04	0.07
Physical status (ASA 3)	0.53	0.31	0.92	0.02
IHD	0.40	0.18	0.86	0.02
Atrial/Ventricular fibrillation	2.95	1.26	6.89	0.01
Anticoagulants	1.22	0.12	12.97	0.87

Note: The reference group for age group variable is “<80”. The reference group for physical status is “ASA 1/2”.

## Data Availability

The datasets generated during and/or analysed during the current study are available from the corresponding author on reasonable request.
